# DKK1 drives immune suppressive phenotypes in intrahepatic cholangiocarcinoma and can be targeted with anti‐DKK1 therapeutic DKN‐01

**DOI:** 10.1111/liv.15383

**Published:** 2022-09-15

**Authors:** Edward J. Jarman, Marta Horcas‐Lopez, Scott H. Waddell, Stephanie MacMaster, Konstantinos Gournopanos, Daniel Y. H. Soong, Kamila I. Musialik, Panagiota Tsokkou, Minn‐E Ng, William A. Cambridge, David H. Wilson, Michael H. Kagey, Walter Newman, Jeffrey W. Pollard, Luke Boulter

**Affiliations:** ^1^ MRC Human Genetics Unit Institute of Genetics and Cancer, University of Edinburgh Edinburgh UK; ^2^ MRC Centre for Reproductive Health Queen's Medical Research Institute, The University of Edinburgh Edinburgh UK; ^3^ Department of Clinical Surgery University of Edinburgh, Little France Crescent Edinburgh UK; ^4^ Leap Therapeutics Cambridge Massachusetts USA

**Keywords:** cholangiocarcinoma, Dickkopf‐1, immune tolerance, macrophage, regulatory T cell

## Abstract

**Background and aims:**

Dickkopf‐1 (DKK1) is associated with poor prognosis in intrahepatic cholangiocarcinoma (iCCA), but the mechanisms behind this are unclear. Here, we show that DKK1 plays an immune regulatory role in vivo and inhibition reduces tumour growth.

**Methods:**

Various in vivo GEMM mouse models and patient samples were utilized to assess the effects of tumour specific *DKK1* overexpression in iCCA. *DKK1*‐driven changes to the tumour immune microenvironment were characterized by immunostaining and gene expression analysis. *DKK1* overexpressing and damage‐induced models of iCCA were used to demonstrate the therapeutic efficacy of DKK1 inhibition in these contexts using the anti‐DKK1 therapeutic, DKN‐01.

**Results:**

*DKK1* overexpression in mouse models of iCCA drives an increase in chemokine and cytokine signalling, the recruitment of regulatory macrophages, and promotes the formation of a tolerogenic niche with higher numbers of regulatory T cells. We show a similar association of DKK1 with FOXP3 and regulatory T cells in patient tissue and gene expression data, demonstrating these effects are relevant to human iCCA. Finally, we demonstrate that inhibition of DKK1 with the monoclonal antibody mDKN‐01 is effective at reducing tumour burden in two distinct mouse models of the disease.

**Conclusion:**

DKK1 promotes tumour immune evasion in iCCA through the recruitment of immune suppressive macrophages. Targeting DKK1 with a neutralizing antibody is effective at reducing tumour growth in vivo. As such, DKK1 targeted and immune modulatory therapies may be an effective strategy in iCCA patients with high DKK1 tumour expression or tolerogenic immune phenotypes.

**Lay Summary**

DKK1 can promote tumour growth and metastasis. It has previously been associated with poorer outcomes in patients with cholangiocarcinoma, a cancer of the bile ducts, tubes that radiate through the liver and normally drain bile into the bowel. In this study, we found that DKK1 changes the tumour microenvironment by altering immune cells that normally act against tumours. By disabling these anti‐tumour cells, DKK1 promotes tumour growth. We found that in mice with cholangiocarcinoma, inhibiting DKK1 reduced tumour growth. Targeting DKK1 represents a therapeutic opportunity for patients with DKK1 expressing tumours.

## 1 INTRODUCTION

Cholangiocarcinoma (CCA) is a group of cancers of the biliary tree, a network of ducts that drain bile into the intestine. Typically described as either intrahepatic (iCCA) or extrahepatic (comprising of distal dCCA and perihilar pCCA), CCAs comprise a highly proliferative epithelium and dense, immune cell‐rich stroma.^1^, ^2^, ^3^ These malignancies constitute approximately 15% of primary liver cancers.^4^ Following diagnosis, patient outcomes are dismal and treatment options are extremely limited, with less than one‐third of patients being eligible for surgery.^4^, ^5^, ^6^ Currently, the majority of patients receive palliative chemotherapy.^7^ In recent years, research has focussed on molecular profiling of CCA and the development of targeted therapeutics such as mutant‐IDH and FGFR inhibitors. These targeted approaches have shown promising results in clinical studies but their application is limited to a small proportion of patients with suitable genetic profiles.^8^, ^9^, ^10^, ^11^, ^12^, ^13^, ^14^ Immune‐directed therapies offer novel and widely applicable potential treatments for CCA, yet clinical trials are in their infancy^15^, ^16^ and to date have had variable success.^4^, ^7^ Importantly, results of the recently published TOPAZ‐1 trial,^17^ which evaluated the efficacy of Durvalumab (an anti‐PD‐L1 agent) and chemotherapy vs chemotherapy alone in advanced biliary tract cancers, demonstrated a promising improvement in survival for patients receiving the anti‐PD‐L1 antibody. Suggesting that immunotherapeutic approaches still hold promise for the disease. CCA, therefore represents a cancer with a patient group who would substantially benefit from the identification of novel therapeutic strategies including immunomodulatory approaches.

Dickkopf‐1 (DKK1) is a secreted WNT signalling modulator which has been shown to be highly expressed in around a third of iCCAs and is associated with worse prognosis.^18^ Interestingly, iCCA is a cancer in which the canonical WNT signalling pathway is activated,^19^ suggesting that DKK1 may not be fulfilling its classical role of preventing WNT receptor activation in these tumours.^20^ Alternative DKK1 functions have been described^21^, ^22^ and increasing evidence suggests an immunological role for DKK1 in cancer.^3^, ^21^, ^22^, ^23^, ^24^ Notably, DKK1 drives the recruitment of myeloid‐derived suppressor cells to the tumour microenvironment^25^ and reduces activation of natural killer (NK) cells,^26^ implicating DKK1 in the modulation of an immunosuppressive tumour microenvironment. Secreted DKK1 can be specifically targeted with DKN‐01 a neutralizing antibody that has been shown to reduce cancer growth in models of both melanoma and prostate cancer in an NK cell‐dependent manner.^24^, ^26^ DKN‐01 is being investigated as a monotherapy or combination therapeutic for various malignancies^27^ and a retrospective analysis suggested that patients with elevated tumoral expression of DKK1 were the more likely to derive clinical benefit from a DKN‐01 anti‐PD‐1 combination therapy.^28^ As such, DKK1 tumoral expression is currently being investigated prospectively as a patient stratification strategy as part of a phase 2 clinical study (NCT04363801). In biliary tract cancers (including iCCA) DKN‐01 used in combination with standard of care chemotherapy (Gemcitabine/Cisplatin) has been shown to be well tolerated but provides no improvement over Gemcitabine/Cisplatin alone (NCT02375880).^29^ Circulating biomarker analysis in this study suggested that DKN‐01 might be immune modulatory, transiently increasing inflammatory cytokines IFNγ, IL6 and IL8; however, DKK1 tumoral expression data were available for only a limited number of patients in this study so the potential benefit of stratification is unclear.

Further understanding of the mechanisms governing DKK1's immune modulatory and tumour‐promoting activity in iCCA is required to provide a rationale for patient stratification or therapy combinations in which DKK1 inhibition can be levied in the most effective way. Using in vivo models of iCCA, we define the effects of high DKK1 expression on the tumour immune microenvironment and test the efficacy of DKK1 neutralization on tumour growth.

## 2 MATERIALS AND METHODS

### 2.1 Animal work

All animal work was performed under the UK Home Office project licence held by Dr Luke Boulter (PFD31D3D4) or Prof Jeffrey Pollard (P9C3F6964). Animals were maintained in colonies in 12 h light–dark cycles and were allowed access to food and water ad libitum.

### 2.2 Hydrodynamic tail vein injection

Female, FVB/N mice were purchased from Charles River, UK and were used at 4–6 weeks of age. Animals were injected with a physiological saline solution (10% w/v) containing naked DNA plasmids into the lateral tail vein in <7 s to achieve a hydrodynamic transfection of the liver. Typical injections contained 6 μg of the sleeping beauty plasmid PGK‐SB13 and a combination of model‐specific plasmids. *Nicd*/*Akt* model: 4 μg pT3‐myr‐*Akt*‐HA (Addgene #31789) and 20 μg pT3‐EF1a‐*Nicd*1 (Addgene #46047). *Kras* ^*G12D*^/g*Trp53* model: 20 μg pCAGGS‐*Kras* ^*G12D*^‐IRES‐GFP (provided by Dr Diego Calvisi, University of Regensberg), 20 μg SB‐CRISPR gRNA containing a pool of three different guides targeting *Trp53* in equimolar quantities (6.6 μg each) (SB‐CRISPR plasmid provided by Prof Dr Roland Rad, LMU Munich).^30^ For *DKK1* overexpression a HA‐tagged human *DKK1* ORF was cloned into pSBbi‐RB (Addgene #60522) using Gibson assembly resulting in DKK1‐HA expression under the control of the EF‐1a promoter, used in HTVI at 20 μg.

### 2.3 Keratin19‐CreERT;*Pten* ^flox^;*Trp53* ^flox^ (KPP) mice

Keratin19‐CreERT (Jackson Labs #026925) mice were crossed with animals containing floxed alleles of *Pten* (Jackson Labs #006440) or *Trp53* (Jackson Labs #008462). Animals heterozygous for Keratin19‐CreERT and homozygous for *Trp53* ^flox^ and *Pten* ^flox^ alleles were used in this study. Mice received three doses of 4 mg of tamoxifen to induce floxed allele recombination, followed by 400 mg/L Thioacetamide in their drinking water. Animals developed well‐differentiated bile duct adenocarcinoma within 8 weeks.^30^

### 2.4 Csf1r‐iCre;Ctnnb1^flox^ and Csf1r‐iCre;Porcn^flox^ mice

Tg(*Csf1r.iCre*)Jwp.‐*Ctnnb* ^fl/fl^ (*Csf1r*‐iCre;*Ctnnb1* ^flox^) mice were generated by crossing B6.129‐Ctnnb1^tm2Kem^ (Jackson Labs #004152) with Tg(Csf1.icre)jwp (Jackson Labs #021024). Tg(*Csf1r*.iCre)Jwp.‐*Porcn* ^tmros^ (*Csf1r*‐iCre;*Porcn* ^flox^) mice have been previously described.^31^ Mice were bred with equivalent Cre‐negative controls (WT). Gene‐targeted mice display myeloid‐specific loss of either *Ctnnb1* or *Porcn* genes. Hydrodynamic injections were performed in these animals using PGK‐SB13, pT3‐EF1a‐*Nicd* and pT3‐myr‐*Akt*‐HA plasmids as described above.

### 2.5 Blood monocyte sorting

Blood was taken from *Csf1r‐iCre;Ctnnb1* ^*flox*^ or *Csf1r‐iCre;Porcn* ^*flox*^ animals. After red blood cell lysis (Biolegend #420301), cells were blocked with 1 μl Fc block/1 x 10^6^ cells/100 μl at 4°C for 15 min. Cells were stained for 30 min at 4°C with antibodies for immune markers CD45, CD11b, CD115, LY6C, LY6G, CD45R/B220, CD49b and CD3 (antibodies are described in detail in Table S1). Cells were sorted using a BD FACS Aria II Flow Cytometer with the addition of DAPI as a live/dead cell marker. Gating for CD115+ monocytes was performed as described in Figure S4.

### 2.6 mDKN‐01 treatment

A murine version of the monoclonal antibody DKN‐01 (mDKN‐01)^24^ was provided by Leap Therapeutics. Mice were dosed twice a week with 20 mg/Kg mDKN‐01 or vehicle control via I.P. injection.

### 2.7 Xenografts of human CCA cell lines

Stable *DKK1* or *GFP* overexpressing CC‐LP‐1 cells were generated by lentiviral transduction of CMV‐DKK1‐HA or CMV‐GFP‐HA genetic constructs into CC‐LP‐1 cells. For xenografts, 4 × 10^5^ cells were re‐suspended in 100 μl of a 1:1 solution of DMEM and matrigel and subcutaneously injected into each flank of athymic CD‐1 Nude mice (Charles River). Starting from 1 week post‐injection, mice received 10 mg/kg DKN‐01 antibody therapeutic or IgG isotype control antibody by I.P. injection twice a week for 5 weeks. Tumour size was assessed by calliper measurement twice weekly and final tumour weights were taken at the end of the experiment.

### 2.8 Histology and Immunohistochemistry

Livers were flushed with saline and lobes were dissected into 10% neutral buffered formalin for 24 hrs. Fixed tissue was processed into paraffin blocks. For immunohistochemistry 4 μm sections were dewaxed in xylene and rehydrated. Following antigen retrieval, samples were incubated with 3% hydrogen peroxide and blocked for avidin and biotin (Abcam, ab64212) followed by a pan‐species protein block (Abcam, ab64226). Primary antibodies were incubated overnight and detected using species‐specific biotinylated secondary antibody and HRP‐DAB detection. Slides were either counterstained with Harris haematoxylin in the case of DAB staining or stained in haematoxylin and eosin (H&E) for assessing tissue histology. Antibodies are listed in Table S1.

### 2.9 Tissue microarray

Slides containing samples from tissue microarray (TMA) LVC1261 (Pantomics) were used for immunohistochemical analysis following the standard IHC protocol described above. The TMA contained 126 cores comprised of two tumours cores and one normal adjacent tissue core from 42 patients with iCCA.

### 2.10 Tumour burden and immune quantification in tissue sections

Tissue slides were scanned using the Nanozoomer slide scanner (Hamamatsu) using a 40× objective and imported into QuPath digital pathology software for analysis. Tumour tissue was annotated, and tumour burden was calculated as a percentage area of the total liver section. Positive cell detection was performed automatically in QuPath after the manual setting of thresholds for cell detection and positive staining. For H‐score determination, thresholds were set for weak, moderate and strong positive staining and applied equivalently across tissue cores. H‐score was determined by multiplying the percentage of cells in the core by their positivity score; 0 = negative, 1 = weak positive, 2 = moderate and 3 = highly positive cells, giving a score range of 0–300.

F4/80 quantification in tumours from *Csf1r‐iCre;Ctnnb1* ^*flox*^ *and Csf1r‐iCre;Porcn* ^*flox*^ mice was performed based on co‐staining for F4/80 and CK19. Sections were imaged using an AxioScan.Z1 slide scanner (Zeiss) and were analysed using Definiens TissueStudio and Developer XD 2.7 software (Definiens Inc.). Tumour‐associated F4/80‐positive cells were defined by an initial detection and expansion of CK19‐positive marker regions to cover intratumoural areas. F480‐positive cells were classified within CK19‐positive areas.

### 2.11 Isolation of RNA

RNA was extracted from 50 to 100 mg of fresh frozen tissue using TRIzol RNA Isolation Reagent (Invitrogen) and was homogenized in a Qiagen TissueLyser LT (QIAGEN). RNA was isolated with chloroform and the RNeasy Mini Kit (QIAGEN) per the manufacturer's instructions.

### 2.12 Reverse transcription and quantitative PCR

Reverse transcription of 1 μg of RNA was performed using Quantitect Reverse Transcription Kit (Qiagen) per the manufacturer's instructions. cDNA was diluted 1:10 and qPCR was performed using the Roche Lightcycler 480 II instrument and Lightcycler 480 Sybr Green Master Mix (Roche) following the manufacturer's instructions with target specific primers used at 10 μM. A full list of primers used can be found in Table S2.

### 2.13 Nanostring gene expression analysis

RNA was isolated as described above. RNA quality was assessed using the Agilent 2100 Bioanalyzer. Nanostring gene expression analysis was performed using the PanCancer IO 360 panel. Data were quality controlled and analysed using the nCounter Advanced Analysis 2.0 software. Gene lists used for Nanostring pathway analysis are shown in Table S3.

### 2.14 Statistics

All statistical analysis was performed in Graphpad Prism 9 unless otherwise stated. In the case of normally distributed data (determined by Shapiro–Wilk testing) unpaired Student's *t*‐test was used. With non‐normally distributed data, the non‐parametric Mann–Whitney test was applied. Pearson's Rank test and associated p‐values were calculated in R.

## 3 RESULTS

### 3.1 *DKK1* overexpression modulates chemokine and cytokine signalling in intrahepatic cholangiocarcinoma

**FIGURE 1:**
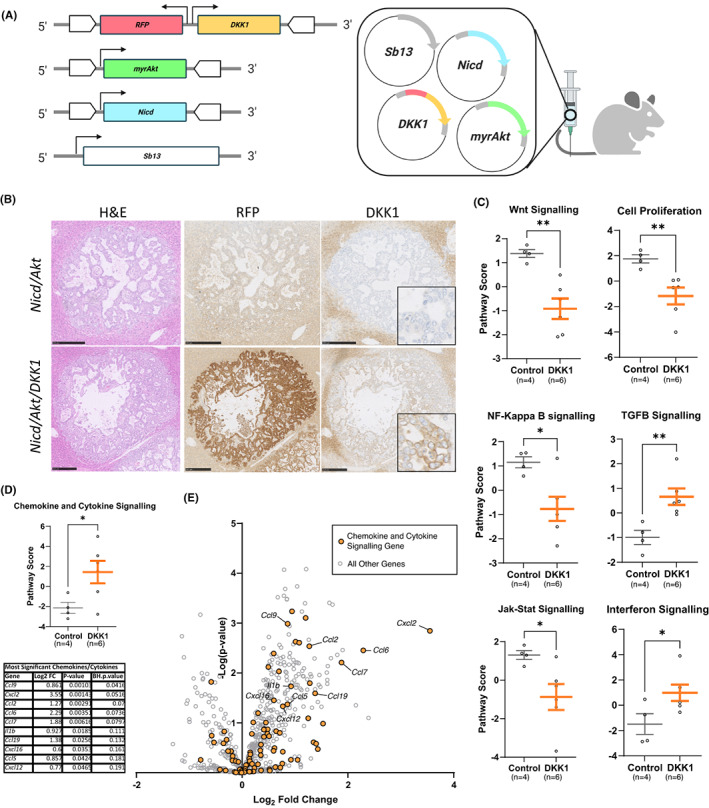
DKK1 overexpression modulates chemokine and cytokine signalling in vivo. (A) Schematic of plasmids used to drive intrahepatic cholangiocarcinoma (iCCA) in our *Nicd*/*Akt* model, retrotransposase IR/DR regions are shown as white block arrows surrounding regions which are thereby integrated into the genome. (B) Histological sections of livers showing individual tumours from our *Nicd*/*Akt* model with and without the DKK1 plasmid included. H&E stained sections (left). Immunohistochemically stained sections of RFP (expressed on the same plasmid as DKK1) (middle), and DKK1 (right) (scale bar: 250 μm). (C) NanoString gene expression data showing combined gene expression scores for relevant pathways in *Nicd*/*Akt* and *Nicd*/*Akt*/*DKK1* tumours, mean pathway scores with SEM are shown. (D) NanoString gene expression score for chemokine and cytokine signalling pathways in *Nicd*/*Akt* and *Nicd*/*Akt*/*DKK1* tumours. A table showing log fold change and p‐value of specific factors is shown below. *p*‐values were corrected for FDR using the Benjamini–Hochberg (BH) procedure and are also shown. (E) Volcano plot showing differential gene expression in *DKK1* samples compared to control. Genes associated with chemokine and cytokine signalling are coloured orange.

Previous human studies have shown that high *DKK1* expression correlates to significantly poorer survival in patients with iCCA.^18^ To investigate the role of high *DKK1* expression on tumour progression, we modified a hydrodynamic tail vein injection model (HTVI) to overexpress *DKK1* in tumour cells. HTVI utilizes high‐volume injections of naked DNA plasmids and the SB13 retrotransposase to promote DNA integration into hepatocytes.^32^ Here, we drive the formation of iCCA by expression of constitutively active myristoylated *Akt* (myr*Akt*) and the Notch intracellular domain (*Nicd*) as previously described.^33^, ^34^ Tumour‐specific overexpression of *DKK1* was achieved by co‐injecting a plasmid expressing both HA‐tagged *DKK1* and *RFP* (Figure 1A)^35^ and allows us to lineage trace *DKK1* overexpressing cells. Livers were excised from mice bearing either *Nicd*/*Akt* or *Nicd*/*Akt*/*DKK1* expressing tumours and contained numerous iCCAs. Tumours in both groups displayed well‐differentiated ductular morphology, high CK19 expression and low levels of AFP (Figure S1). High expression of DKK1 was observed specifically in tumours in the *Nicd/Akt/DKK1* group (Figure 1B; Figure S1). Gene expression analysis showed a significant reduction in the activation of WNT signalling and cell proliferation pathways when *DKK1* was overexpressed (Figure 1C). However, *DKK1* expression induced numerous gene expression changes associated with modulation of the immune signalling, including a significantly lower activation of the inflammatory NF‐κB and JAK–STAT signalling pathways, as well as an upregulation of the immunosuppressive TGFβ and interferon signalling pathways (Figure 1C). *DKK1* also drove a significant increase in chemokines and cytokines in these tumours (Figure 1D) including *Ccl* and *Cxcl* family members that are involved in the recruitment of various immune cell types (Figure 1E). The highest fold change was seen in *Cxcl2* expression (>10‐fold, *p* = .0014), with a greater than 2‐fold change in gene expression seen in *Ccl2*, *Ccl6*, *Ccl7* and *Ccl19*. This analysis identified alterations in immune modulatory pathways as a key differential between *DKK1* overexpressing and control tumours in this model.

### 3.2 DKK1 recruits immune suppressive myeloid cells to the tumours

**FIGURE 2:**
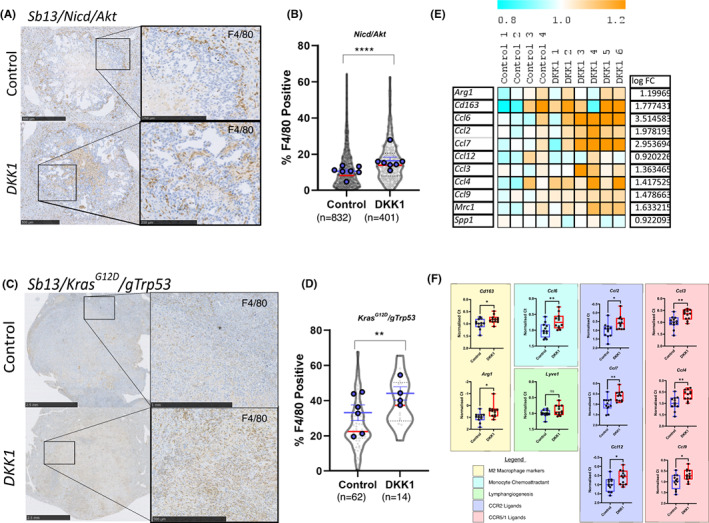
DKK1 promotes the recruitment of F4/80 TAM2 macrophages to tumours. (A) Immunohistochemistry of F4/80 in *Nicd*/*Akt* (top) or *Nicd*/*Akt*/*DKK1* (bottom) tumour bearing livers (scale bars = 500 μm [insets = 250 μm]). (B) Quantification of F4/80‐positive cells shown in A as a percentage of total cell count within tumour regions. Individual tumour measurements shown in grey (violins with red median line) (*p* < .0001, unpaired Student's *t*‐test), while average measurements per animal are superimposed in blue with mean and SEM shown. (C) Immunohistochemical staining of F4/80 in *Kras* ^*G12D*^/g*Trp53* tumours with and without DKK1 overexpression (scale bars = 2.5 mm [inset top = 1 mm, inset bottom = 500 μm]). (D) Quantification of F4/80 staining in *Kras* ^*G12D*^/g*Trp53* model. Positive cells are shown as a percentage of total cells within the tumour region. Individual tumours shown in grey (violins with red median line) (*p* = .0013, unpaired Student's *t*‐test), while average measurements per animal are superimposed in blue with mean and SEM shown. (E) Heat map representation of normalized gene expression values for TAM2 associated genes from the NanoString gene expression experiment in *Nicd*/*Akt* (control) and *Nicd*/*Akt*/*DKK1* (DKK1) tumours. (F) Quantitative real time PCR expression showing a similar increase in TAM2 associated markers in *Kras* ^*G12D*^/g*Trp53* tumours when *DKK1* is overexpressed (*n* = 12 vs 12). Changes are shown as Ct values normalized to housekeeping gene expression.

Due to the association of DKK1 with both immune modulation in our in vivo model and worse outcomes in patients with iCCA,^18^, ^24^, ^25^, ^26^ we sought to explore whether *DKK1* expression promotes immune cell recruitment to the iCCA tumour microenvironment. We made use of our *Nicd/Akt* model (detailed above) and a second genetic model, where the expression of mutant *Kras* ^*G12D*^ and genetic deletion of *Trp53* with CRISPR‐Cas9 in vivo (*gTrp53*) results in *Kras* ^*G12D*^‐driven cholangiocarcinoma.^30^ Despite higher expression of *Cxcl2* (Figure 1E), we saw no increase in tumour neutrophil recruitment (determined by percentage of Myeloperoxidase‐positive cells) when *DKK1* was overexpressed in *Nicd*/*Akt* or *Kras* ^*G12D*^ */gTrp53* tumours (Figure S2). In *DKK1*‐overexpressing *Nicd/Akt*‐driven iCCA we found an increase in the proportion of F4/80‐positive cells from 10.5% in control tumours (*n* = 832), to 15.58% in tumours that overexpressed *DKK1* (*n* = 401) (*p* < .0001) (Figure 2A and B). In addition, we observed a similar increase in F4/80‐positive cells in *Kras* ^*G12D*^ */gTrp53* tumours overexpressing *DKK1* (Figure 2C and D). In this second model, the proportion of F4/80 cells was increased from an average of 25.94% (*n* = 62) in the control group up to 39.39% (*n* = 19) of cells when *DKK1* was overexpressed in tumour cells (*p* = .0013).

To define the nature of tumour‐associated F4/80‐positive cells in these models, we looked at co‐expression of F4/80 with macrophage polarization markers CD163 (a classical pro‐inflammatory marker) and CD68 (a classical pro‐restorative marker) in our *Nicd/Akt*‐driven model with and without the overexpression of *DKK1*. In both contexts, the majority of F4/80‐positive cells infiltrating the tumour were negative for both CD68 and CD163 (Figure S3). This suggests that recruited macrophages may be naïve or poorly polarized. To better understand the nature of these cells we performed gene expression analysis using markers previously shown to be specifically upregulated in MHCII^low^ immunosuppressive TAM2 macrophages.^36^ These genes demonstrated an overall increase when *DKK1* is overexpressed in our models (Figure 2E and F) compared to control tumours. These genes include markers of alternative macrophage polarization (*Arg1*, *Cd163*), monocyte chemoattractant *Ccl6*, and ligands for CCR2 (*Ccl2*, *Ccl7*, *Ccl12*) and CCR5/1 (*Ccl3*, *CCl4*, *Ccl9*) suggesting that F4/80+ cells in these tumours represent an increase in TAM2‐like myeloid cells. Interestingly, we were able to demonstrate that this is not a consequence of DKK1‐mediated WNT signalling inhibition in myeloid cells themselves. Using transgenic mouse models for the myeloid‐specific deletion of β‐catenin (*Csf1r‐iCre/Ctnnb1* ^*flox/flox*^) required for canonical WNT signalling or Porcupine (an O‐acetyltransferase required for WNT ligand processing and therefore production) (*Csf1r‐iCre/Porcn* ^*flox/flox*^) we induced iCCA formation over 6 weeks using hydrodynamic injection of *Nicd*/*Akt* (Figure S4). Using these mouse models, we were able to validate effective loss of *Porcn* or *Ctnnb1* in monocytic cells, and this did not affect the circulating numbers of CD115+ monocytes in the blood (Figure S4A,B) of either *Csf1r‐iCre/Ctnnb1* ^*flox/flox*^ or *Csf1r‐iCre/Porcn* ^*flox/flox*^ mice. Furthermore, myeloid cell ablation of either *Porcn* or *Ctnnb1* had no effect on tumour formation in the *Nicd*/*Akt* iCCA model (Figure S4C). Consistent with these data on tumour growth, loss of *Porcn* or *Ctnnb1* did not result in a change in the recruitment of F4/80‐positive macrophages to tumour regions (Figure S4D) nor of FOXP3+ regulatory T recruitment (Figure S4E). These data collectively show that neither loss of macrophage WNT production nor reception are sufficient to recapitulate immune phenotypes produced by *DKK1* overexpression.

### 3.3 *DKK1* overexpression promotes a tolerogenic immune microenvironment

**FIGURE 3:**
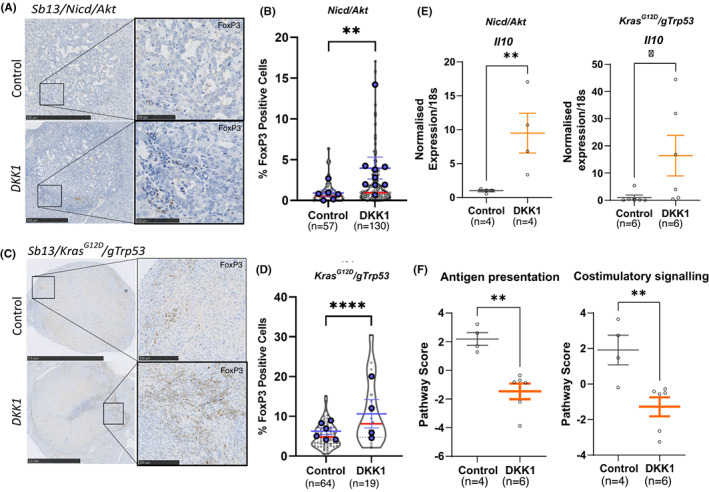
DKK1 overexpression promotes the formation of a tolerogenic immune microenvironment in cholangiocarcinoma (CCA). (A) Immunohistochemical staining for FOXP3 (brown) in *Nicd*/*Akt*‐driven cancers with and without *DKK1* expression (scale bars = 500 μm [insets = 250 μm]). (B) Quantification of FOXP3 staining in *Nicd*/*Akt* and *Nicd*/*Akt*/*DKK1* tumours (*n* = 57 vs 130). Positive cells are shown as a percentage of total cells in the tumour area (violin plots with red median lines) (*p* = .0076, unpaired Student's *t*‐test), while average measurements per animal are superimposed in blue with mean and SEM shown. (C and D) Equivalent data to A and B above for the tumours in the *Kras* ^*G12D*^/g*Trp53* HTVI model (scale bars = 2.5 mm [insets = 250 μm]) (*n* = 64 vs 19) (*p* < .001, unpaired Student's *t*‐test). (E) Real time qPCR of *Il10* expression from tumours in the *Nicd*/*Akt* and *Kras* ^*G12D*^/g*Trp53* models. Points show relative expression values normalized to 18 s RNA expression in control and DKK1 overexpressing tumours, bars represent mean values and SEM for *n* = 4 vs 4 tumours (*Nicd*/*Akt*, *p* = .0017) and *n* = 6 vs 6 (*Kras* ^*G12D*^/g*Trp53*, *p* = .0107). (F) NanoString gene expression data showing combined gene expression scores for antigen presentation and costimulatory signalling, bars represent the mean and SEM (*p* = .0014 and *p* = 0.0093 respectively).

Having established a *DKK1*‐driven increase in TAM2‐like macrophages in our hydrodynamic models, we next sought to explore whether *DKK1* expression can promote tolerogenicity in the iCCA tumour microenvironment. We looked at the number of tumour associated FOXP3+ regulatory T cells (*T*_reg_) located within *DKK1* overexpressing and control tumours (Figure 3A‐D). *T*_reg_ are highly immunosuppressive and their abundance has been associated with worse prognosis in a number of cancers.^37^, ^38^, ^39^ When *DKK1* was overexpressed in our *Nicd*/*Akt* model, we found a significant increase in FOXP3+ cells within tumour boundaries (*p* = .0076). On average 2.07% of cells were FOXP3 positive when *DKK1* was highly expressed (*n* = 130), compared to 0.933% in control tumours (*n* = 57) (Figure 3B). A similar increase in FOXP3+ cells was found in our *Kras* ^*G12D*^/g*Trp53* model (Figure 2C and D). When *DKK1* was overexpressed in this tumour model the proportion of FOXP3‐positive *T*_reg_ was increased from an average of 5.3% (*n* = 64) in the vector control group up to 10.7% (*n* = 19) of cells (*p* < .001).

In addition to FOXP3 cell number, both *Nicd*/*Akt* and *Kras* ^*G12D*^/g*Trp53* models showed increased expression of the immune suppressive interleukin *Il10* when *DKK1* was overexpressed (*p* = .0017, fold change = 9.49 and *p* = .0107, fold change = 16.41, respectively, Figure 3E). Finally, the fact that *DKK1* is inducing a tolerogenic immune microenvironment in these tumours was supported by Nanostring gene expression data, which confirmed that *Nicd*/*Akt*/*DKK1* tumours have reduced gene signatures associated with antigen presentation and costimulatory signalling compared to *Nicd*/*Akt* alone (*p* = .0014 and *p* = .0093 respectively) (Figure 3F).

**FIGURE 4:**
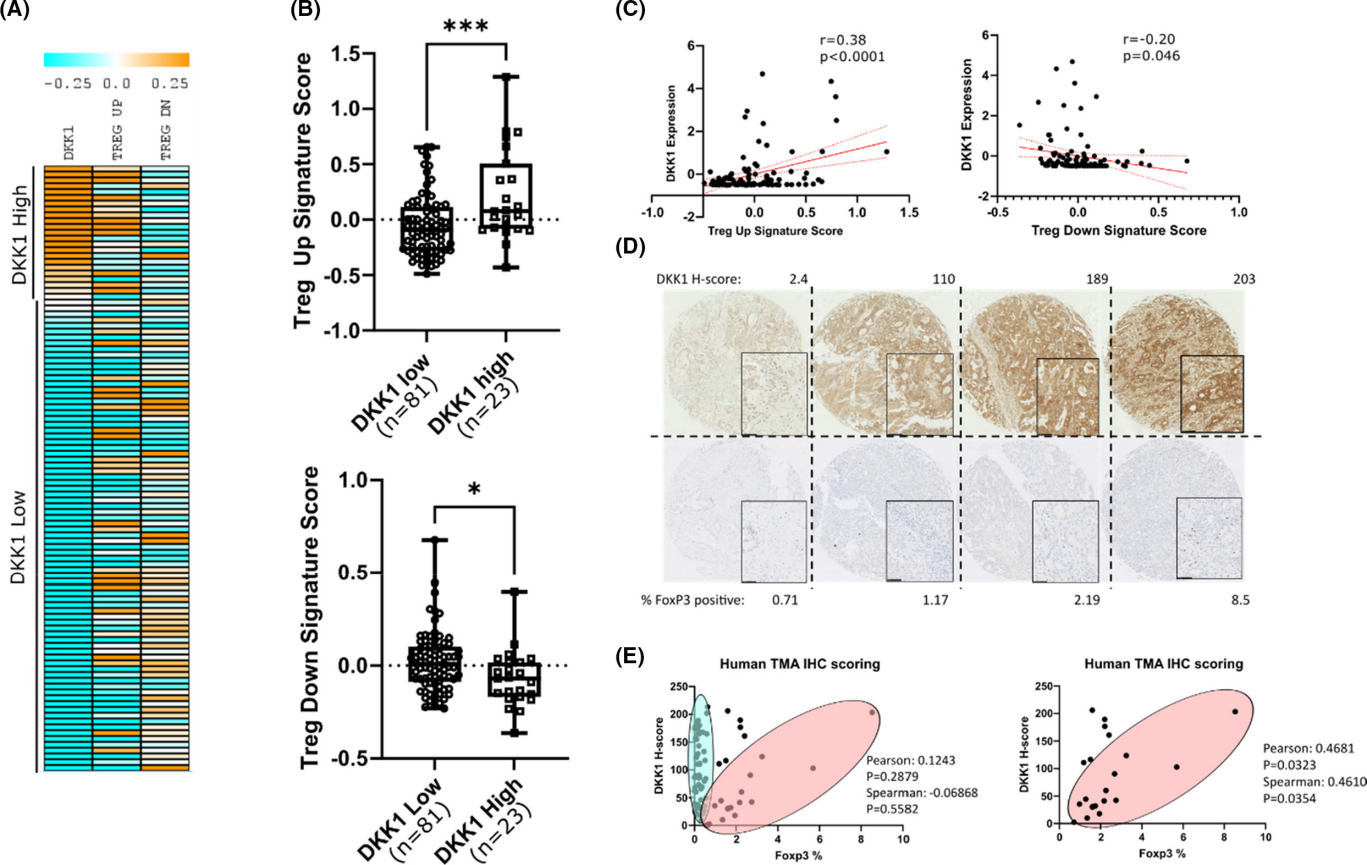
DKK1 is associated with increased FOXP3 regulatory T cells in human cholangiocarcinoma (CCA). (A) Heat map representation of gene expression in patient intrahepatic cholangiocarcinoma (iCCA) samples ordered by mean centred expression values for *DKK1*, which are shown alongside averaged gene scores for gene signatures upregulated in *T*_reg_ (Treg up) and downregulated in *T*_reg_ (Treg down). (B) Box and whisker plots showing mean centred gene expression values of TREG up (Top) and TREG down (Bottom) gene expression signatures in patients stratified into high and low groups based on *DKK1* expression. Boxes represent the median and interquartile ranges of *n* = 81 vs 23 patients in *DKK1* low and high groups respectively. *p* values were obtained by unpaired *t*‐test (*p* = .0003 and .0195 respectively). (C) Scatter plots showing mean centred, quantile normalized gene expression values for *DKK1* vs gene expression scores for signatures associated with *T*_reg_ upregulation (Treg up) and downregulation (Treg down) in data taken from Illumina beadchip expression array of CCA samples (GSE26566). Each point represents a separate patient sample (*n* = 104), linear regression line is shown in solid red (95% confidence intervals are red dotted lines). Pearson's correlation co‐efficient (*r*) and *p*‐values for significant correlation are shown. (D) Immunohistochemical staining of tumour cores from a tissue microarray (TMA) of iCCA patients stained with DKK1 (top) or FOXP3 (bottom) showing representative examples from low to high expression (scale bars = 50 μm). (E) Scatterplots showing the relationship between DKK1 H‐score and FOXP3% positivity from immunohistochemistry of the TMA. Patients appear to separate into two populations represented by blue and red ovals. While no significant correlation is seen in the population as a whole, when patients with the lowest FOXP3 values (blue oval) are removed (% FOXP3 < 0.7) (these patients display negligible levels of FOXP3 staining but a high variability in DKK1) the remaining population (red oval) demonstrates positive correlation between DKK1 and FOXP3 protein levels.

To support evidence from our in vivo models, we assessed whether DKK1 might recruit FOXP3 regulatory T cells in human iCCA by analysing transcriptomic Illumina beadchip array data from 104 cholangiocarcinomas (GSE26566) (Figure 4A–C). These data were interrogated for expression of *T*_reg_ transcriptional signatures.^40^, ^41^ When stratified by mean *DKK1* expression (*DKK1* high *n* = 23, *DKK1* low *n* = 81) (Figure 4B), genes associated with *T*_reg_ were significantly higher in *DKK1‐*high patients when compared to the *DKK1‐*low group (*p* = .0003) (top), and genes with reduced expression during *T*_reg_ induction were significantly lower in the *DKK1* high (*p* = .0195) (bottom). Additionally, these signatures show significant correlation with *DKK1* expression across the entire 104 patient cohort (Figure 4C); positive correlation was seen between the average expression of *T*_reg_‐induced genes and *DKK1* expression (*r* = 0.38, *p* < .0001), while negative correlation was seen between *DKK1* expression and *T*_reg_ downregulated genes (*r* = −0.20, *p* = .046). Having defined a relationship between *DKK1* and *T*_reg_ signatures in iCCA, we used a tissue microarray to assess the number of FOXP3+ cells in iCCA patient tissue and compared this to DKK1 protein levels from the same tumours (Figure 4D and E). A large number of patient cores were essentially devoid of FOXP3 cells with no correlation to DKK1 levels (blue oval, Figure 4E). However, in patients with higher FOXP3 cell abundance (>0.7% FOXP3 cells, red oval Figure 4E) we saw a correlation between FOXP3 cell number and DKK1 protein level (*r* = 0.4681, *p* = .0323, *n* = 21).

### 3.4 Anti‐DKK1 therapeutic mDKN‐01 reduces tumour burden in pre‐clinical models of iCCA

**FIGURE 5:**
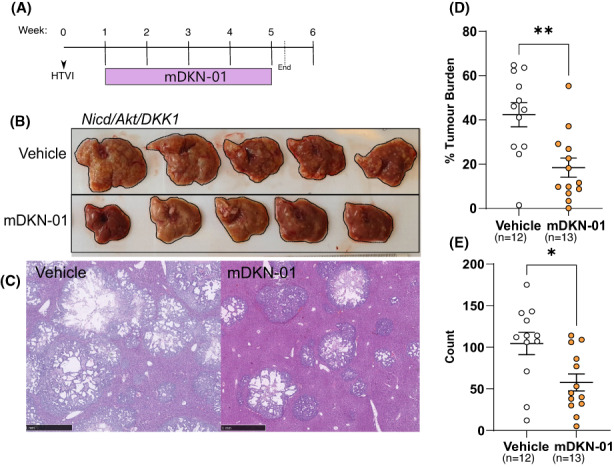
DKK1 inhibition with mDKN‐01 reduces tumour burden in *Nicd*/*Akt*/*DKK1* mice. (A) Schematic of *Nicd*/*Akt*/*DKK1* tumour bearing mice treated with mDKN‐01. (B) Whole livers of mice in vehicle group (top) or mDKN‐01 (bottom). (C) H&E staining showing representative examples of liver tumour burden in vehicle‐treated (left) and mDKN‐01‐treated (right) liver (scale bars = 1 mm). Quantification of liver burden (D) and tumour count (E) data from vehicle or mDKN‐01‐treated mice, individual mice are represented by points, bars show the mean and SEM. P‐values were obtained through unpaired Students *t*‐tests (*p* = .0022 (burden) and *p* = .0104 [count]).

Having established a role for DKK1 in promoting tumour immune modulation and defined the relationship between DKK1 levels and FOXP3+ cells in a cohort of human samples, we next wanted to test whether DKK1 inhibition was effective at reducing iCCA growth. Following *Nicd*/*Akt*/*DKK1*‐driven tumour initiation, mice were treated with an anti‐DKK1 neutralizing antibody (mDKN‐01) or vehicle control for 4 weeks (Figure 5A). Representative livers and H&E stains are shown in Figure 5B and C respectively. In these mice, we found a significant reduction in both tumour burden and the number of individual tumours within the liver when treated with mDKN‐01 (Figure 5D and E). Mean liver tumour burden was reduced from 42.36% in vehicle‐treated animals (*n* = 12) to 18.45% in the mDKN‐01 treatment group (*n* = 13) (*p* = .0022) (Figure 5D). Similarly, tumour number was reduced from an average of 104 tumours per section in the vehicle group down to 58 following treatment (*p* = .0104) (Figure 5E). While mDKN‐01 shows demonstrable efficacy in this immune‐competent model, we see no reduction in tumour growth as a result of DKN‐01 treatment in human CCA cell lines when xenografted into athymic CD‐1 nude mice (Figure S5). This loss of efficacy was seen with both tumours from CC‐LP‐1 cells expressing endogenous levels of DKK1 as well as from CC‐LP‐1 genetically modified to overexpress the protein, and demonstrates that the mechanism of action for anti‐DKK1 monoclonal therapeutics requires an intact adaptive immune system.

**FIGURE 6:**
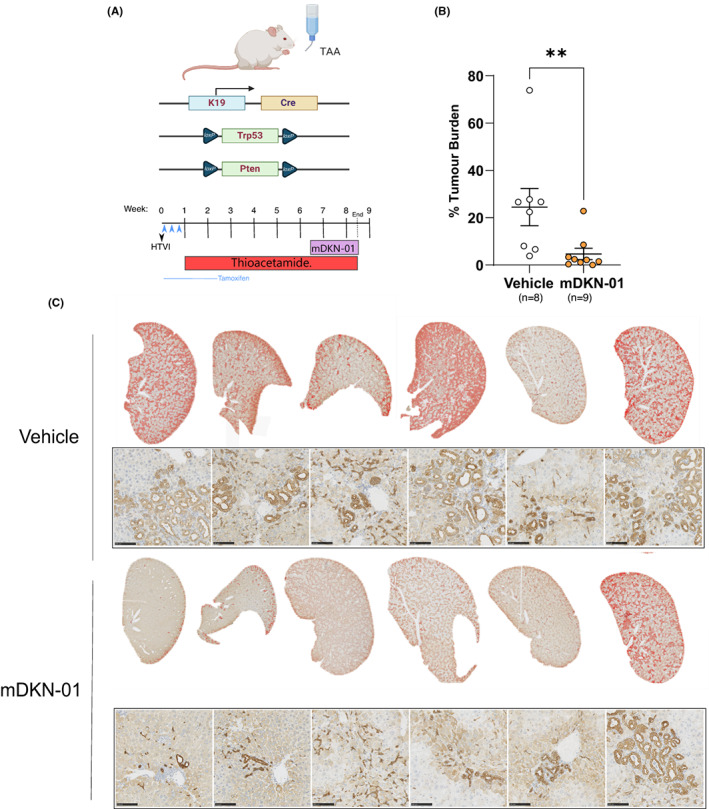
DKK1 inhibition is effective in a damage‐induced model of intrahepatic cholangiocarcinoma (iCCA). (A) Schematic representation of experimental set‐up in our Keratin19‐CreERT/*Pten* ^flox/flox^/*Trp53* ^flox/flox^ thioacetamide model (KPP) of iCCA. Mice were treated with tamoxifen to induce Keratin19‐Cre‐driven recombination of floxed *Trp53* and *Pten* genes specifically in cholangiocytes. Mice then received 400 mg/L TAA in their drinking water for 7.5 weeks. Mice received I.P. injections 20 mg/kg mDKN‐01 or vehicle control twice a week for 2 weeks. (B) Quantification of tumour burden in liver sections from these animals showing significantly reduced tumour coverage in mDKN‐01‐treated mice (*p* = .0037 Mann–Whitney). (C) Histological representation of tumour burden in vehicle and mDKN‐01‐treated livers. Top rows show low magnification liver sections with Keratin‐19‐positive areas overlaid in red to highlight differences in tumour coverage between these mice. Representative regions showing Keratin‐19 staining and tumour histology at higher magnification are shown below (scale bars = 100 μm).

Finally, we wanted to define whether mDKN‐01 shows efficacy in a more pathologically relevant, damage‐induced model of iCCA where tumours develop on the background of chronically inflamed liver with endogenous levels of DKK1, thereby better reflecting human disease aetiology. To do this, we used a recently published transgenic model where *Trp53* and *Pten* are deleted specifically in the biliary epithelium (by Keratin19‐CreERT, herein referred to as the KPP model) following the administration of Tamoxifen (Figure 6A).^30^ When combined with thioacetamide (TAA) induced liver damage, mice develop diffusely distributed, well‐differentiated iCCA within 8 weeks. KPP mice were dosed with mDKN‐01 or vehicle control twice weekly for the final 2 weeks before the end of the experiment (Figure 6A). We found that mDKN‐01 was able to reduce tumour burden in the liver from a mean of 24.5% in the vehicle control (*n* = 8) down to 4.7% in mDKN‐01‐treated mice (*n* = 9) (*p* = .0037) (Figure 6B). (Representative images of tumour burden is shown by CK19 immunohistochemistry, Figure 6C). This suggests that the inhibition of DKK1 could be an effective strategy at reducing tumour growth in a more physiologically and clinically relevant model with an active inflammatory driver, and without the artificial overexpression of *DKK1*.

## 4 DISCUSSION

DKK1 has been shown to be overexpressed and associated with worse outcomes in a number of cancers^42^ and in addition to WNT signalling pathway modulation, DKK1 may affect tumour immunity through direct actions on NK cells or MDSCs.^24^, ^25^, ^26^ High DKK1 levels are seen in approximately one‐third of iCCA patients but the effects of DKK1 expression on the immune microenvironment of iCCA is not known.^18^ By using genetically tractable mouse models of iCCA to understand the effect of *DKK1* overexpression on the tumour immune microenvironment, we have shown that *DKK1* is sufficient to drive profound immune changes within tumours. Having defined how DKK1 modulates this system, we demonstrate that therapeutic targeting of DKK1 can drastically reduce tumour size and number in both hydrodynamic and damage‐induced mouse models of iCCA.

In two genetically distinct hydrodynamic models of iCCA, *DKK1* expression resulted in increased recruitment of FOXP3+ *T*_reg_ that can drive immunosuppression through a number of competitive and non‐competitive mechanisms.^38^, ^43^, ^44^, ^45^, ^46^, ^47^ Unsurprisingly, *T*_reg_ abundance has been associated with worse prognosis in a number of cancers.^37^, ^38^, ^39^ *T*_reg_ recruitment to the tumour microenvironment is dependent on a range of chemokines and cytokines^48^ which are typically produced by macrophages or tumour cells in the tumour niche. In our models of iCCA, we found a robust increase in F4/80+ cells when *DKK1* is overexpressed and a concomitant increase in the expression of TAM2 associated factors, suggesting that increased FOXP3+ cells may be a secondary result of DKK1‐induced TAM2 recruitment. Notably, CCR5 ligands *Ccl3* and *Ccl4* are upregulated when *DKK1* is expressed and have previously been shown to be strong mediators of *T*_reg_ infiltration in pancreatic adenocarcinoma.^49^

Previous work by D'Amico et al. have shown that in mammary carcinoma DKK1 promotes the accumulation of MDSCs,^25^ which are a heterogeneous population of immature myeloid cells with low levels of F4/80. While these findings differ from our findings in iCCA, it is salient to note that myeloid lineages are complex and fluid in cancer, and that MDSCs can differentiate into regulatory TAMs depending on tumour context.^36^, ^50^, ^51^ Importantly, both MDSCs and TAMS can be immunosuppressive and result in the formation of a tolerogenic immune environment.

Importantly, we have shown, both through immunohistochemical analysis of patient tissue and transcriptional changes associated with Treg upregulation, that a relationship between DKK1 and regulatory T cells also exists in human iCCA samples. Previous studies looking at DKK1 levels in patients with CCA have found no association of DKK1 levels with liver cirrhosis, viral hepatitis or NASH.^18^, ^52^ This suggests that the association between DKK1 levels and Treg abundance is unlikely to be due to a role for DKK1 expression in a common underlying inflammatory pathology, and that mechanisms of DKK1‐driven immune suppression may be relevant to a subset of DKK1 high patient iCCAs.

Finally, DKK1 appears to drive an immunosuppressive microenvironment associated with intrinsic or acquired PD‐1 resistance (upregulation of TGFβ, downregulation of JAK signalling and upregulation of regulatory macrophages). We have shown that uncoupling DKK1 from its immune regulatory network by treatment with mDKN‐01 is effective at reducing tumour growth in multiple models of iCCA. In addition, mDKN‐01 has been shown to have efficacy in other pre‐clinical models of cancer, and an early phase I trial of DKN‐01 with gem/cis in biliary tract cancer has demonstrated that the drug is well tolerated. Our data indicate that these studies would benefit from patient stratification depending on the DKK1 status of the cancer.

## FUNDING INFORMATION

This study was partly funded by Leap Therapeutics and by Cancer Research UK (grant number C52499/A27948). M.H.L. was funded by the Wellcome Trust (108 906/Z/15/Z). J.W.P. was funded by a Wellcome Trust Senior Investigator Award (101 067/Z/13/Z) W.A. Cambridge was funded by The Royal College of Surgeons of Edinburgh.

## CONFLICT OF INTEREST

This study was partially funded by Leap Therapeutics. M.K. and W.N. are employees and stockholders and/or stock option holders of Leap Therapeutics Inc. JWP is a founder, shareholder in and on the board of Macomics Ltd an immuno‐oncology company. These studies, however, do not conflict with those of the company. All other authors have no further conflicts to disclose.

## Patient consent statement

All patient data used in this study was fully anonymised.

## Supporting information


Data S1
