# Supplementary material for: DKK1 drives immune suppressive phenotypes in intrahepatic cholangiocarcinoma and can be targeted with anti‐DKK1 therapeutic DKN‐01
Source: Liver Int. 2022 Sep 15;43(1):208–20. doi: 10.1111/liv.15383 (PMC10087034; doi:10.1111/liv.15383)
Supplement: Supplementary file 1 — Data S1 [file LIV-43-208-s001.docx]

**Supplementary material**

**Title: DKK1 drives immune suppressive phenotypes in intrahepatic cholangiocarcinoma and can be targeted with anti-DKK1 therapeutic DKN-01**

**Authors:**

**Edward J. Jarman^1,#^**

**Marta Horcas-Lopez^2^**

**Scott H. Waddell^1^**

**Stephanie MacMaster^1^**

**Konstantinos Gournopanos^1^**

**Daniel Y.H. Soong^2^**

**Kamila I. Musialik^1^**

**Panagiota Tsokkou^1^**

**Minn-E Ng^1^**

**William A. Cambridge ^1,3^**

**David H. Wilson^1^**

**Michael H. Kagey^4^**

**Walter Newman^4^**

**Jeffrey W. Pollard^2^**

**Luke Boulter^1,#^**

**Affiliations:**

1. MRC Human Genetics Unit, Institute of Genetics and Cancer, University of Edinburgh, United Kingdom.
2. MRC Centre for Reproductive Health, Queen's Medical Research Institute, The University of Edinburgh, Edinburgh, United Kingdom.
3. Department of Clinical Surgery, University of Edinburgh, Little France Crescent, Edinburgh, United Kingdom.
4. Leap Therapeutics, Cambridge, Massachusetts, USA.

**Contents**

1. **Supplementary table 1: Antibodies used in this study**
2. **Supplementary table 2: PCR primers used in this study**
3. **Supplementary table 3: Nanostring pathway score gene lists**
4. **Supplementary figure 1**
5. **Supplementary figure 2**
6. **Supplementary figure 3**
7. **Supplementary figure 4**
8. **Supplementary figure 5**

**Supplementary table 1: Antibodies used in this study**

**Supplementary table 2: PCR Primers used in this study**

**Supplementary table 3: NanoString gene lists used for pathway scoring**

**Supplementary Figure 1:**


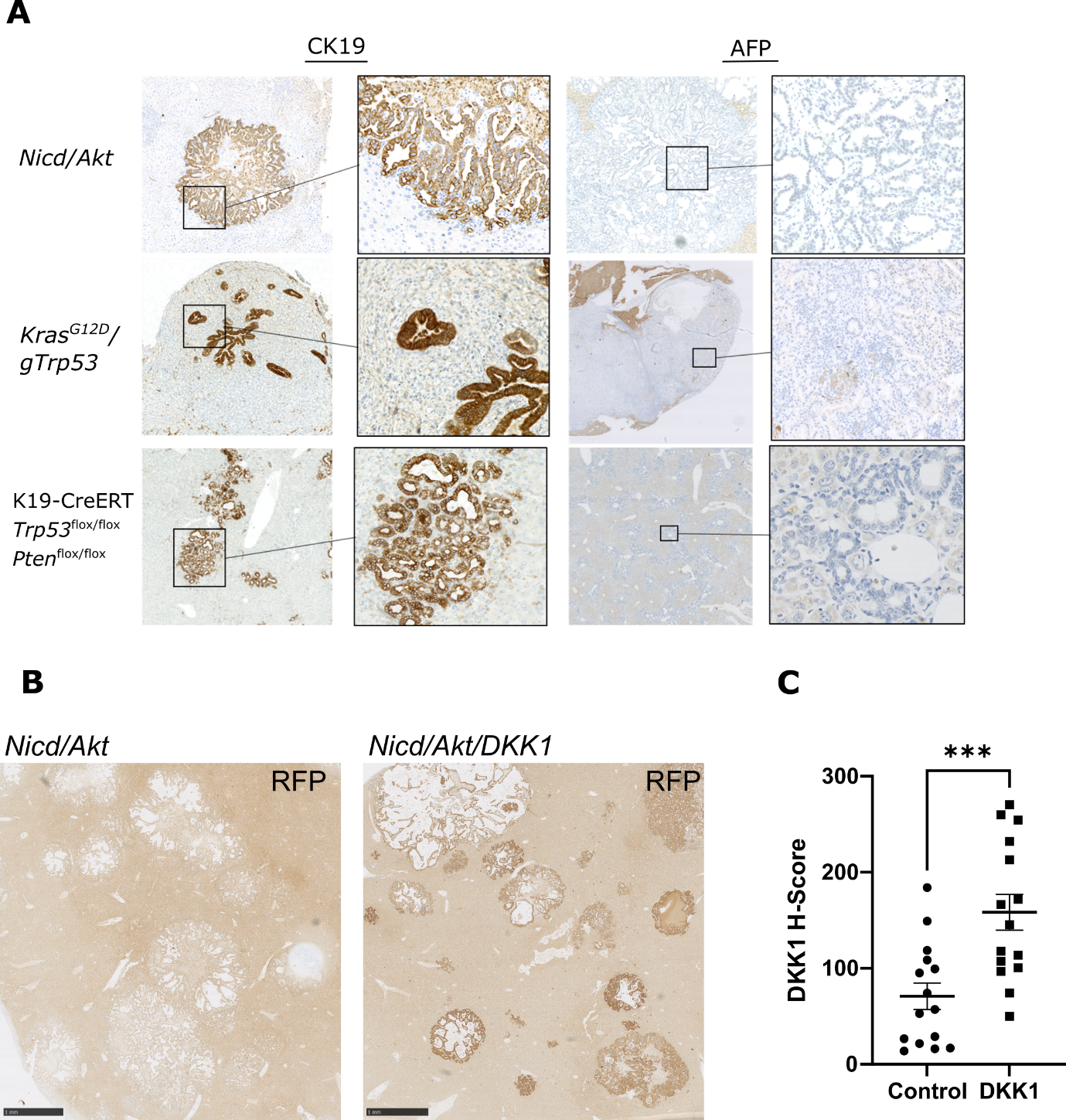


**Supplementary Figure 1: Representative histology of mouse models used in this study.**

A) Representative staining of Keratin-19 (CK19) and Alpha fetoprotein (AFP) in *Nicd/Akt* and *Kras*^G12D^/g*Trp53* hydrodynamic injection models, as well as in our thioacetamide induced Keratin19-CreERT *Pten/Trp53* floxed model (KPP) of intrahepatic cholangiocarcinoma. All three models show generally high staining of CK19 and low/undetectable staining of AFP. *Kras*^G12D^/g*Trp53* shows a more desmoplastic phenotype with more variable staining for CK19 and some areas of moderate AFP. B) Comparison of RFP staining in *Nicd/Akt* hydrodynamic tumours with and without ectopic *DKK1* (RFP) expression. This demonstrates successful expression of this construct in this model, and the expected tumour-specific expression pattern. C) Immunohistochemical scoring of DKK1 expression in the *Nicd/Akt* hydrodynamic model, with and without overexpression of *DKK1*. Whilst control tumours do show endogenous levels of DKK1 (n=15), expression is significantly increased when the *DKK1* plasmid is incorporated (n=15) (p=0.0008, unpaired Student’s T-test).

**Supplementary figure 2:**


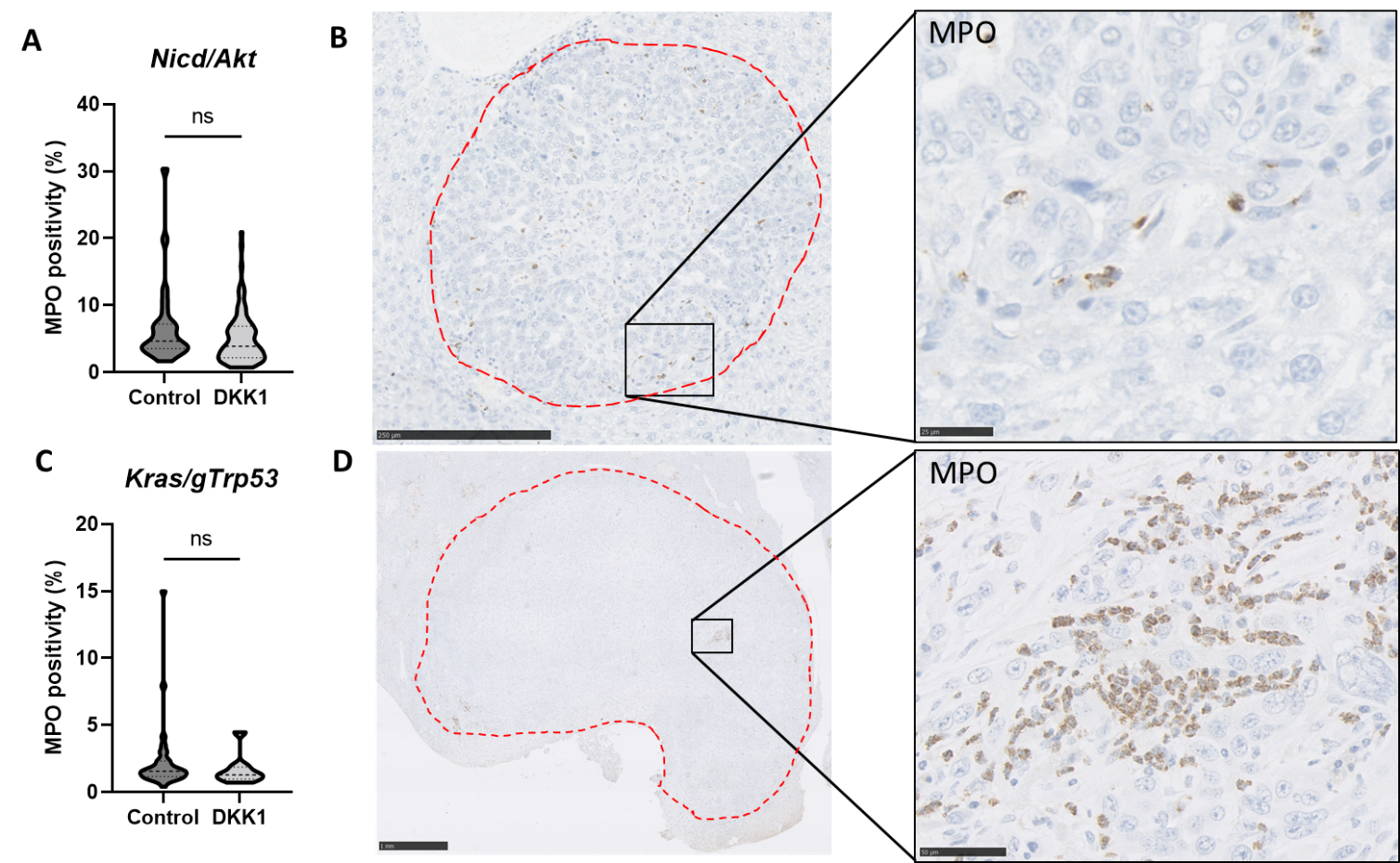


**Supplementary figure 2: No change in tumour neutrophil recruitment when *DKK1* is overexpressed.**

Neutrophil recruitment was assessed by myeloperoxidase (MPO) staining in *Nicd/Akt* and *Kras^G12D^/gTrp53* tumour models and compared with and without the overexpression of *DKK1*. A) Quantification of MPO positive cells as a percentage of total cells within tumour regions of *Nicd/Akt (n=26)* and *Nicd/Akt/DKK1 (n=89)* tumours. B) Example of MPO DAB staining in a *Nicd/Akt* tumour (scale bars = 250µm (inset = 25µm)). C) Quantification of the percentage of MPO positive cells with tumour regions in *Kras^G12D^/gTrp53 (n=33)* and *Kras^G12D^/gTrp53/DKK1 (n=9)* tumours (scale bars = 1mm (inset = 50µm)). D) Example image of MPO DAB staining in a *Kras^G12D^/gTrp53* tumour.

**Supplementary figure 3:**

**
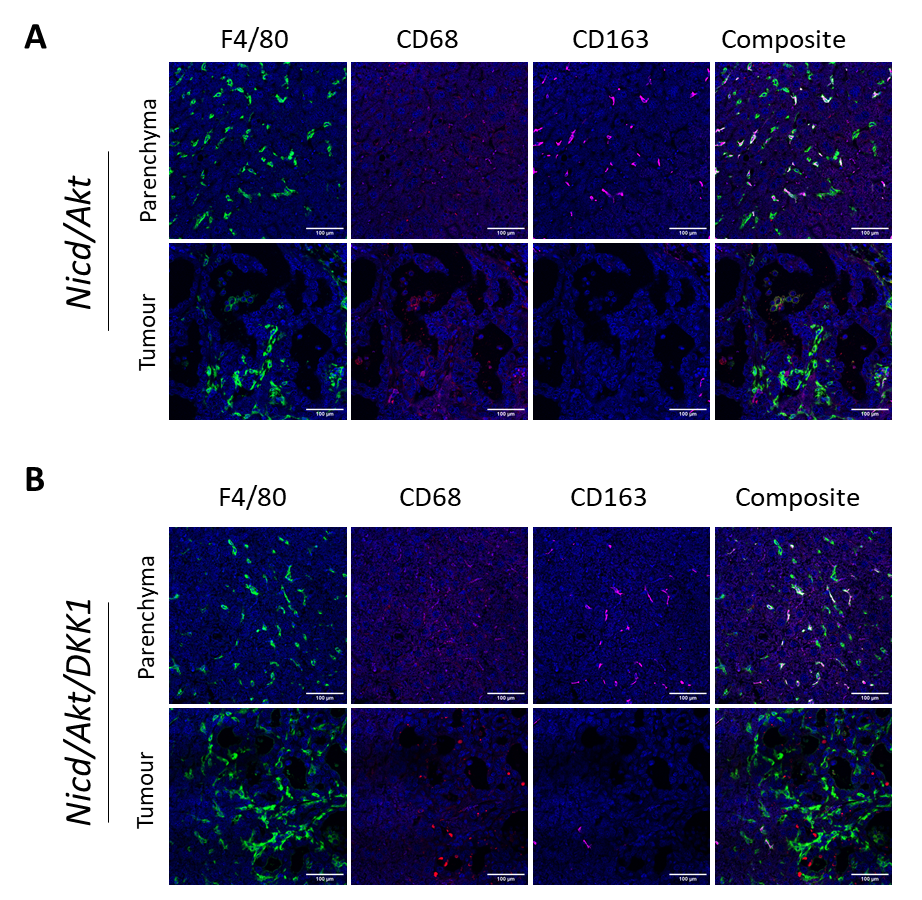
**

**Supplementary figure 3: Tumour infiltrating F4/80 positive cells do not express CD163 or CD68 polarisation markers.**

Co-immunofluorescent characterisation of CD163 (magenta) and CD68 (red) in F4/80 (green) positive cells (scale bars = 100µm). A) Examples of macrophage marker staining in normal liver parenchyma (top row) and within tumour regions (bottom row). Parenchyma shows clear presence of CD163 positive Kupffer cells. Tumours are largely devoid of CD163 positive cells and have small numbers of CD68 positive cells. The majority of F4/80 positive cells stain for neither of these markers. B) Equivalent staining in DKK1 overexpressing tumours (Parenchyma (top) and Tumour regions (bottom)). There is no clear difference in the representation of CD163 and CD68 markers in *Nicd/Akt* tumour when *DKK1* is overexpressed.

**Supplementary figure 4:**


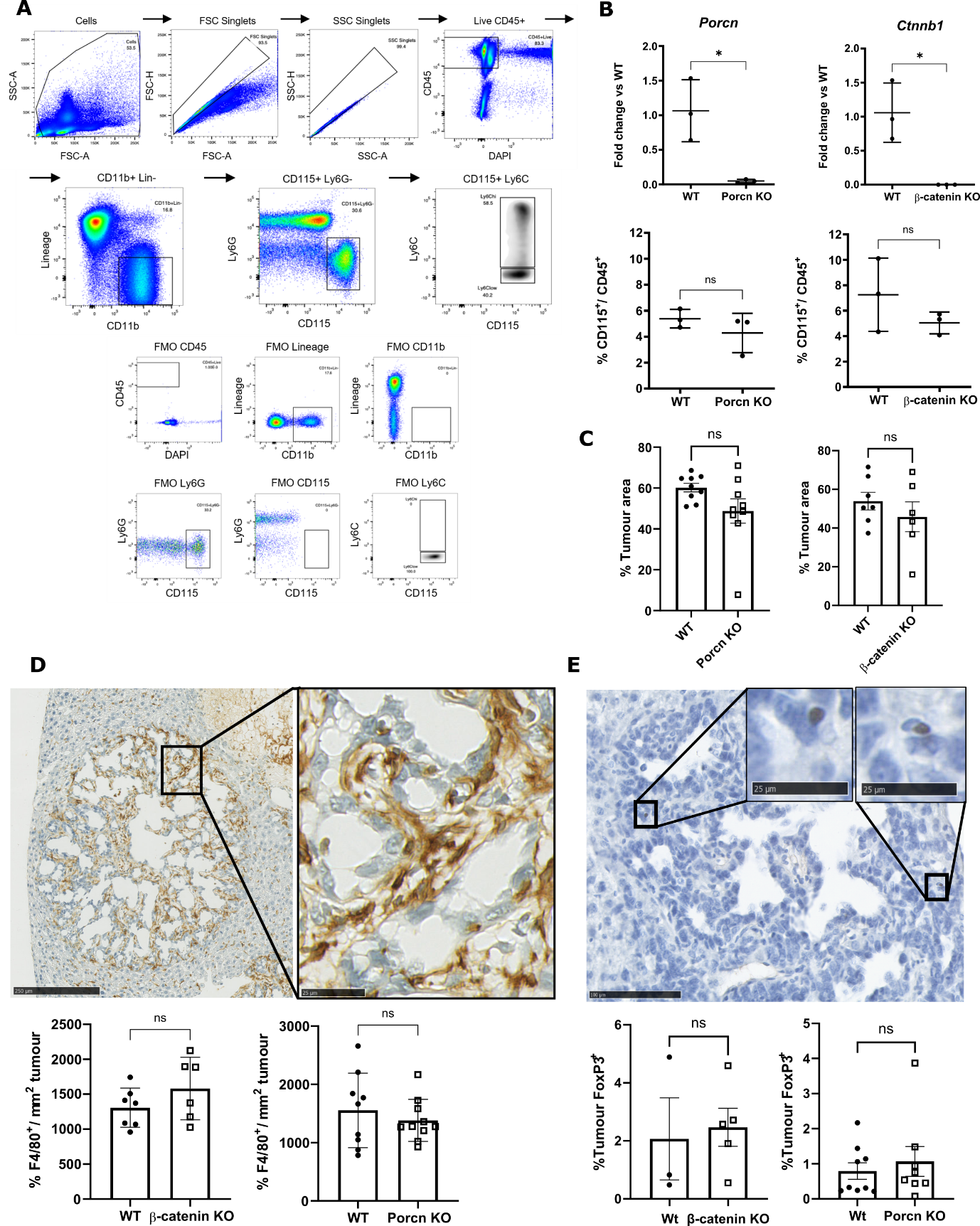


**Supplementary Figure 4: The DKK1 immune phenotype is not driven by WNT inhibition in myeloid cells.**

Use of mouse models for the myeloid-specific deletion of β-catenin (*Csf1r-iCre/Ctnnb1^flox/flox^*) or Porcupine (an O-acetyltransferase required for WNT ligand processing;*Csf1r-iCre/Porcn^flox/flox^*) to assess whether loss of WNT signalling or production in myeloid cells can recapitulate any of the DKK1 phenotypes in *Nicd/Akt* driven tumours. A) Example of the FACS gating for the isolation of CD115+ monocytes from peripheral blood from *Porcn* or *Ctnnb1* before and after gene ablation. B) Top graphs: Complete loss of *Ctnnb1* or *Porcn* gene expression in CD115+ monocytic cells isolated from *Csf1r-iCre* mice (n=3) in comparison with wild-type animals (n=3). Gene expression was measured by qPCR. Bottom graphs: Loss of *Porcn* or *Ctnnb1* in monocytes did not change their abundance as determined by flow cytometry (n=3 vs 3). C) Histological measurement of tumour burden in Cre-negative (wild-type) and Cre-positive (KO) models for *Ctnnb1 (n=7 vs 6)* and *Porcn (n=9 vs 9)*, 6 weeks after hydrodynamic injection. These data show no change in tumour burden after loss of β-catenin signalling or WNT ligand secretion in myeloid cells when compared to wild-type animals. D) Quantification of F4/80 macrophage numbers in *Nicd/Akt* tumours with and without myeloid-specific loss of key WNT signalling components. The top panels show representative staining of F4/80+ macrophages in these tumours (scale bar =250µm (inset =25 µm)), with quantification of F4/80+ cells below. This is shown as a percentage of total cells in the tumour area between wild-type animals and those with *Ctnnb1 (n=7 vs 6)* or *Porcn (n=9 vs 10)* deletion. No significant difference in F4/80 number was seen. E) Quantification of FOXP3+ regulatory T cell number in *Nicd/Akt* tumours with and without myeloid-specific loss of key WNT signalling components. The top image shows representative staining of FOXP3+ cells (scale bar =100µm (insets =25µm)) with quantification shown below. No significant difference was seen between wild-type and *Ctnnb1 (n=3 vs 5)* or *Porcn* (n=9 vs 8) KO animals.

**Supplementary Figure 5:**


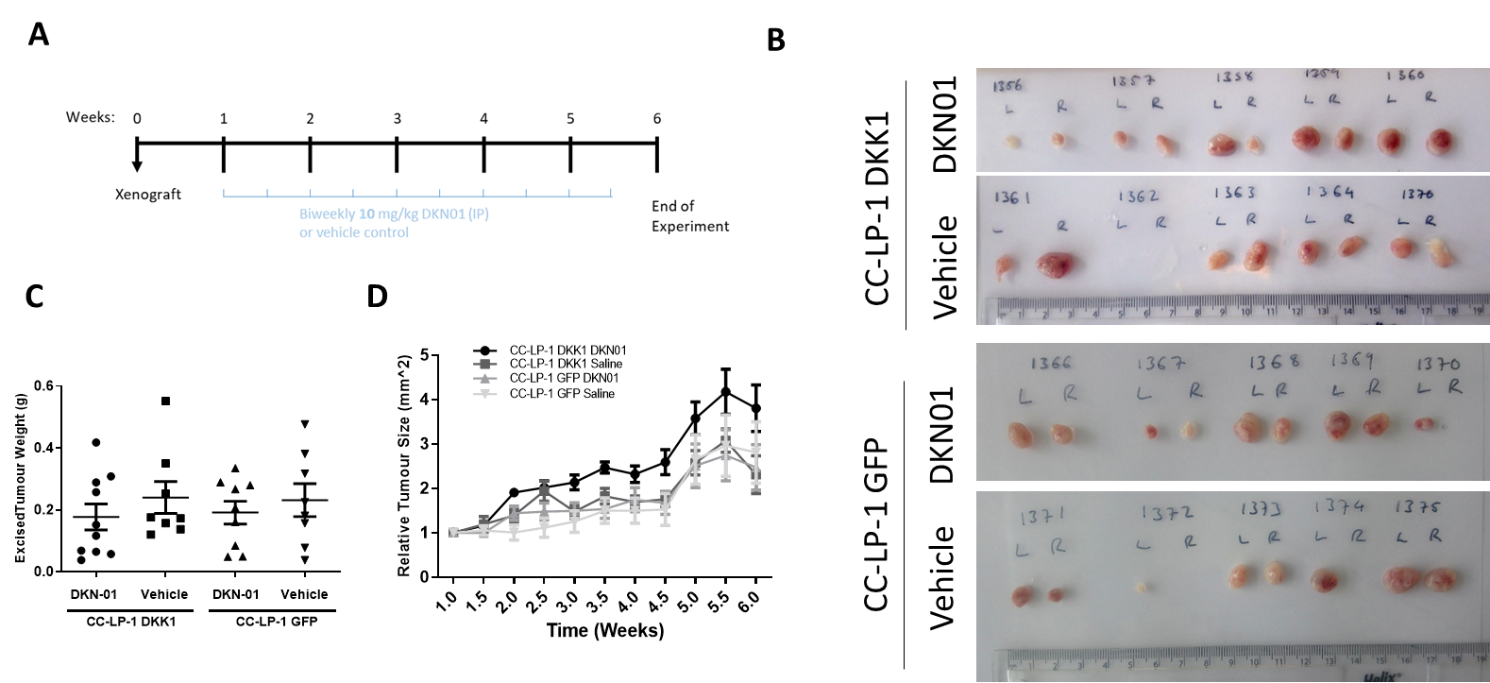


**Supplementary Figure 5: Anti-DKK1 monoclonal antibody DKN-01 does not reduce the growth of human CCA cell lines in an immune compromised xenograft model.**

A) Athymic CD-1 nude mice were injected subcutaneously on both flanks with the human iCCA cell line CC-LP-1 overexpressing either *DKK1* or *GFP*. Mice were then given I.P. injections of 10mg/kg DKN01 or vehicle control. Xenograft growth was assessed by calliper measurements over 6 weeks and final tumour weight after excision. B) Images of excised tumours from left (L) and right (R) flanks of CD-1 nude mice 6 weeks after injection with CC-LP-1 DKK1 cells (top 2 panels) and CC-LP-1 GFP cells (bottom panels). C) Excised tumour weights 6 weeks after subcutaneous injections with CC-LP-1 DKK1 (n=10 (DKN-01) vs n=8 (vehicle)) or CC-LP-1 GFP (n=9 (DKN-01) vs n=8(vehicle)). No significant difference was seen between groups. D) Tumours sizes as measured by bi-weekly calliper measurements relative to the size at week 1. No benefit from DKN-01 was seen in either DKK1 overexpressing or GFP expressing cell lines (n= 10 vs 8 vs 9 vs 8).
